# Supplementary material for: Systematic analysis of non-melanoma skin cancer burden: A comparative study between China and the world from 1990 to 2021 and prediction to 2036
Source: PLoS One. 2025 Dec 5;20(12):e0333960. doi: 10.1371/journal.pone.0333960 (PMC12680255; doi:10.1371/journal.pone.0333960)
Supplement: Attachment — Submitted filename: Response to Reviewers.docx [file pone.0333960.s001.docx]

PLOS ONE

Aug 28, 2025

PONE-D-25-28458

Systematic analysis of non-melanoma skin cancer burden: a comparative study between China and the world from 1990 to 2021 and prediction to 2036

Dear editor and reviewers,

We appreciate editor and reviewers very much for their positive and constructive comments and suggestions on our manuscript entitled “Systematic analysis of non-melanoma skin cancer burden: a comparative study between China and the world from 1990 to 2021 and prediction to 2036.” (Manuscript ID: PONE-D-25-28458). Those comments are all valuable and very helpful for revising and improving our paper, as well as the important guiding significance to our researches. Based on these comments and suggestions, we have made careful modification on the original manuscript.

On the separate pages, we provided our response to the comments and suggestions, point by point, and highlighted the changes in the marked copy of the revision. We hope that our revision will be approved by the experts and reviewed favorably.

Sincerely,

Juan Mei Cao, MD

**Reviewer #1:** Title: Systematic analysis of non-melanoma skin cancer burden: a comparative study between China and the world from 1990 to 2021 and prediction to 2036.
I. Overall
1. Abbreviations should be defined at first mention and used consistently thereafter (eg. UV, etc.).

**Response：**Thank you very much for your careful review. We have revised the full text.
II. Abstract
1. Objective: Kindly enunciate the objective through the use of an action verb.

**Response：**Thank you for your reasonable suggestion. We have made the revisions in lines 4-6: “To compare the characteristics and trends of the non-melanoma skin cancer (NMSC) burden in China and globally, and to provide a basis for the development of effective prevention and control measures in China.”
2. Keywords:
a) Kindly replace "Non-melanoma skin cancer" with "Skin cancer."
b) Although "Bayesian age-period-cohort model" and "Joinpoint regression model" are not directly listed in MeSH, terms like "Bayesian Analysis" and "Regression Analysis" can be suitable alternatives.
c) Kindly replace "Burden of Disease" with "Global Burden of Disease."

**Response：**Thank you for your professional advice. We have modified the key words in lines 29-30: “Skin cancer; Regression analysis; Bayesian analysis; Global burden of disease; Comparative study.”
III. Introduction
1. Lines 30-32 ► “According to GLOBOCAN […] 8% of all cancer deaths”: This declaration is not substantiated by the cited source. It is important to review the statistics you provided to ensure they are supported by a reference.

**Response:** Thank you very much for your professional advice. We are extremely sorry for this error and have checked and re-described it in lines 34-36: “According to GLOBOCAN, there were 1,234,595 new cases and 69,481 deaths of NMSC globally in 2022, accounting for 0.7% of all cancer deaths[2].”
2. Lines 35-36 ► “The age-standardized incidence […] to 784 (/100,000) in 2019 [5]”: This declaration is not substantiated by the cited source. You need to adopt a more meticulous approach when presenting statements and statistics.

**Response:** Thank you for your careful review. We have made the necessary corrections in lines 39-41: “The age-standardized incidence rate (ASIR) of NMSC in the United States had increased from 402 (/100,000) in 1990 to 787 (/100,000) in 2019[5].”
3. Lines 36-39 ► “Moreover, […] in most countries [6]”: I think you can find a more recent reference.

**Response：**Thank you for your reasonable suggestions. We have reviewed and added to, as well as updated, the relevant supporting literature in lines 41-49: “However, the actual incidence of NMSC may be underestimated, potentially due to the following reasons: many countries did not require reporting NMSC information to national cancer registries, which directly led to most countries not giving sufficient attention to NMSC[6-9]; the high incidence of NMSC not only resulted in a large workload and difficulty in implementing comprehensive registration, but also posed technical challenges in accurately recording each patient's tumor status and extracting structured pathological data, making it difficult to obtain relevant data[10]. These factors collectively contribute to the fact that the actual burden of NMSC remains unclear and is frequently underestimated[11].”
4. Lines 40-41 ► “At present, […] clinical treatment [7, 8]”: Regarding the main assertion of this passage, I was convinced that the references you mentioned would be systematic reviews or meta-analyses demonstrating that there are very few epidemiological studies on NMSC conducted in China, which is not the case. The two references you wisely cited fail to support this fundamental claim.

**Response:** We fully agree with your suggestion. We made the necessary revisions in lines 50-52: “Compared with melanoma, NMSC has a lower mortality rate and better prognosis. This difference has led to limited scientific evidence on the epidemiological characteristics and burden of NMSC in China[12].”

5. Lines 42-42 ► “Epidemiological evidence […] interventions”: Isn’t it paradoxical to compare the epidemiological data on NMSC between China and the rest of the world, considering you mentioned in the previous paragraph that there is a lack of data on this type of cancer in China?

**Response:** We fully agree with your point of view. We made revisions in lines 53-60 to make the logic more reasonable: “Therefore, in order to better address the challenges posed by NMSC, this study, based on the Global Burden of Disease (GBD) 2021 database, used the Joinpoint regression model and the Bayesian age-period-cohort (BAPC) model first to analyze and predict the burden (incidence rate, mortality rate, and disability-adjusted life years (DALYs)) caused by NMSC in China from 1990 to 2021 by gender, and then compared it with the global situation, to provide a basis for formulating more effective prevention and treatment strategies and intervention measures.”
6. I believe it would be wise to delve deeper into the epidemiological context of NMSC in China.
**Response:** We fully agree with your opinion and accept it entirely. We have re-examined the relevant literature and revised the introduction section. We are very much looking forward to your approval.

IV. Materials and methods
1. To ensure the reproducibility of the data analysis, please indicate whether model non-identifiability was addressed and specify the evaluation criteria.

**Response:** Thank you for your scientific suggestions. We use the coefficient of determination R^2^ to evaluate the predictive performance of the BAPC model. The larger the R^2^ value, the better the model fits the data. We made corresponding modifications in lines 110-113: “To verify the accuracy of the model, we calculated the coefficient of determination (R²) for each BAPC model. R² reflects the extent to which the model explains the variation in the data. The closer the value is to 1, the better the model fit and the higher the prediction accuracy.”

In lines 145-147: We used the BAPC model to predict the ASIR of NMSC in China and globally based on different genders. The model fit was excellent (R^2^_China male_=0.99998, R^2^_China female_=0.99998; R^2^_Global male_=0.96204, R^2^_Global female_=0.98055).

In lines 177-179: We used the BAPC model to predict the ASMR of NMSC in China and globally based on different genders. The model fit was excellent (R^2^_China male_=0.99643, R^2^_China female_=0.98751; R^2^_Global male_=0.86609, R^2^_Global female_=0.98699).

In lines 210-212: We used the BAPC model to predict the ASDR of NMSC in China and globally based on different genders. The model fit was excellent (R^2^_China male_=0.99987, R^2^_China female_=0.99949; R^2^_Global male_=0.97468, R^2^_Global female_=0.99812).

2. Please provide a reference for the formula you used for the breakpoint regression model.

**Response:** Thank you very much for your suggestion. We have added the references in lines 99:“The model formula was as follows[14]”

V. Results
1. Wouldn’t it be wise to isolate age and gender in Figures 1, 3, and 5?

**Response：**Thank you very much for your suggestion. These three figures are mainly designed to show the trend over time. We believe that combining age and gender can help observe the trend and at the same time avoid presenting more similar figures. We hope you can understand this.
VI. Discussion
1. When the acronym "DALY" is used as a quantifiable measure, it should be employed in the plural form (DALYs).

**Response:** Thank you very much for your careful review. We have made revisions to the entire text.

2. Lines 175-178 ► “Although the level of NMSC […] than that of the world”: This passage must be revised to ensure a balance between precision and fluidity, while also eliminating redundancy and fixing the spelling of the acronym "DLAY."

**Response:** We are grateful for your reasonable suggestions. We have made the necessary changes in lines 225-228: “Although the ASIR of NMSC in China was lower than the global level, the levels of both ASMR and ASDR were relatively higher. Meanwhile, the increased rates of the three indicators for NMSC in China were faster than that in the world.”

3. Lines 178-181 ► “There were significant gender […] faster in women”: To enhance the clarity of this text, it would be appropriate to split it into two distinct sentences, highlighting the parallelism between the incidence comparisons and those of mortality/DALYs, while incorporating a smoother transition between the two sentences.

**Response:** Thank you very much for your patient review. We have made the necessary changes in lines 228-231: “There were significant gender differences in the burden of NMSC: the incidence and its increase rate among men were both higher than those of women; the mortality and DALYs rate for men were more severe, but their increase rates for women were faster.”
4. Lines 204-205 ► “These factors […] from 5th in 1990 to 4th today”: This sentence does not accurately reflect the cited source [20]. It actually outlined the changes in the most common types of cancer and the main causes of cancer-related mortality during the period from 2018 to 2020, rather than from 1990 to 2025, which is implied by your use of the word "today."

**Response:** Thank you very much for your careful review. We have made the necessary corrections in lines 287-288: “These factors combined to increase the incidence of NMSC in China from 5th in 2018 to 4th in 2020[24], a growth rate that exceeded the global average level.”
5. Lines 211-213 ► “Second, China’s medical […] developed coastal areas”:
a. The logical connector "Second" that you used is not appropriate;
b. It is imperative to cite one or more references.

**Response:** We fully agree with your point of view. We have made revisions and additions in lines 291-296: “Although NMSC can usually be cured through surgery, it has been reported that patients with NMSC in China are usually in the middle to late stages when diagnosed, missing the best treatment and having a shorter average survival[28]. Furthermore, China's medical resources are unevenly distributed geographically, with high-quality medical resources more concentrate in large cities and developed coastal areas[29–31].”

6. Lines 216-219 ► “According to […] in men than women [22]”: This passage does not match the indicated reference. The mention of the "American Cancer Society" is inappropriate, as the study was conducted by authors who are individuals. The use of expressions like "twice, three times" to quantify proportions makes it difficult to validate and align with the cited data.

**Response:** Thank you very much for bringing this issue to our attention. We have made revisions and additions in lines 299-313 of the revised version: “This study further found that the incidence of NMSC was higher in men than in women, both globally and in China. This finding was consistent with existing literature reports, with some studies observing similar patterns of gender differences[32,33]. This difference may be related to the different works and lifestyles of men and women. Men worked outdoors more frequently and were, therefore, more exposed to UV light. In addition, men also used sunscreen, hats, and other protective gear less often than women[19]. At the genetic level, males exhibited higher p53 mutation frequencies than females in various cancers, including esophageal cancer, NMSC, and hepatocellular carcinoma. As a key tumor suppressor gene, p53 plays a central role in DNA repair and cell cycle regulation. Higher p53 mutation rates in male skin cells may increase the risk of developing NMSC, accelerate tumor progression, and result in poorer clinical outcomes. This genetic-level gender difference may provide an important molecular biological explanation for the higher burden of NMSC in men compared to women, and also reflected the foundational influence of gender on cancer susceptibility and development[34].”

7. Lines 224-228 ► “Firstly, […] of developing cancer”: The use of the past tense in writing this passage deceives the reader; it is phrased in a way that makes it seem like a conclusion, while it actually represents aspects of the discussion.

**Response:** We completely agree with you. We made the modifications in lines 314-321: “The possible reason for this could be that human skin is in prolonged contact with the natural environment and exposed to sunlight, thus becoming prone to aging as one gets older[2]. At the same time, the immune system of the elderly is weaker, and their immune function is weakened. Abnormal cells cannot be completely eliminated and gradually proliferate, thereby increasing the risk of cancer. This emphasizes the importance of attention and preventive measures for the elderly[35,36].”
8. Lines 237-240 ► “Between […] by 6.4% per year”:
a. Firstly, this passage does not match the indicated reference [26];
b. Secondly, if it turns out that this passage is supported by a reference, I believe that is misplaced, particularly as it comes after a discussion on the Chinese context, even though it emphasizes the American context.

**Response:** Thank you very much for bringing this issue to our attention. We have made revisions and additions in lines 328-339: “Between 2013 and 2017, the introduction of immune checkpoint inhibitors and targeted therapies significantly reduced melanoma mortality rates in the United States[38–40]. Similar therapeutic advancements were gradually applied to NMSC. For example, the PD-1 inhibitor Cemiplimab had been approved by the FDA for advanced cutaneous squamous cell carcinoma, with an objective response rate of up to 47%[41]; the Hedgehog pathway inhibitor Vismodegib achieved an objective response rate of 43% in advanced basal cell carcinoma[42]. Access to these therapies in China is gradually improving, such as the clinical trials of the domestically produced PD-1 inhibitor Toripalimab in skin cancer, and the widespread adoption of early diagnostic technologies (such as dermatoscopy and AI-assisted screening)[43–46], which may drive a reduction in mortality rates and disease burden for NMSC in China.”

VII. References
1. Reference [20] has been incorrectly cited in the final list of references.

**Response:** Thank you very much for your question. Modified.
2. It is clear that the references need to be verified, especially since the year of publication is often repeated in the entries.

**Response:** Thank you very much for your question. Modified.
3. It would also be wise to include the DOI when possible.

**Response:** Thank you very much for your question. We have added.

**Reviewer #2:** in conclusion sections we need to be more specific and get specific recommendations for specific geographic area , there is need to get a specific location inside China with high pervelance of malenoma

**Response：**Thank you very much for your professional advice. Our study was conducted based on the GBD database. However, GBD currently does not provide data for various regions in China, so we are currently unable to analyze the burden of NMSC in different regions of China and cannot determine which region is more severely affected. This is the limitation of this study. We have added a description of the limitations in the discussion section, hoping for your understanding, lines 378-381: “Meanwhile, GBD currently lacks data for various regions in China, our study is unable to compare the severity of NMSC in different regions of China and cannot propose regional prevention and control recommendations.”

In addition, we have supplemented the preventive suggestions regarding NMSC in lines 272-281: “Based on this trend, it is recommended to strengthen the early screening and diagnosis system for NMSC, especially in areas with scarce medical resources; conduct health education and regular check-ups for key populations such as the elderly and young women; incorporate NMSC prevention and control into climate change response strategies and strengthen the construction of UV radiation warning systems; optimize the allocation of medical resources to enhance primary healthcare facilities' diagnostic and treatment capabilities; more national and subnational epidemiological studies on NMSC should be conducted to establish prevention and control strategies and clinical guidelines tailored to the national context, providing scientific evidence for public health decision-making.”

**Reviewer #3:**

General comments:
The study is generally well-structured with time trend and prediction components. However, there are a few important statistical limitations and opportunities for enhancement.
1. While BAPC is an appropriate choice, the manuscript does not clearly communicate whether posterior distributions, credible intervals, or model diagnostics (e.g., DIC, convergence checks) were evaluated to assess forecast reliability. The use of national-level aggregates may mask important subnational variations. The authors could consider a Bayesian hierarchical model or spatiotemporal modeling approach (e.g., INLA, STAN) in future work to more precisely estimate and compare disease burden patterns across regions, especially within China. The projections assume the continuation of historical patterns. Incorporating scenario-based forecasts (e.g., demographic shifts, UV index changes, intervention uptake) would make predictions more policy-relevant.
Although this is not a meta-analysis in the strict sense, the paper would benefit from methods used in systematic reviews, such as sensitivity analyses, effect size estimations, and heterogeneity testing, especially when comparing China to global averages.

**Response:** Thank you very much for your scientific opinions and reasonable suggestions for future study. We fully agree with your viewpoint. In the future, more models should be utilized to study the spatio-temporal distribution patterns, especially to conduct a more detailed analysis and comparison of the differences among various regions in China, while simultaneously considering influencing factors such as population structure. This will be a more meaningful research. This study is a secondary analysis based on GBD data. GBD has not yet provided data for more regions in China, so we analyzed the situation in China and compared it with the global situation, which to some extent helps policymakers formulate relevant policies and can serve as a data reference. In the GBD database analysis, we mainly relied on the data and methodological framework that have already been standardized in the database. The GBD study itself has adopted complex statistical models to estimate the disease burden, including uncertainty intervals and covariate adjustments, which to some extent have considered the variability and robustness of the data. Regarding the BAPC model, it has been applied multiple times to disease prediction, and its predictive effect has been recognized. We supplemented R^2^ to verify the model's fitting effect. We have made modifications and additions in lines 105-113: “We used the BAPC model to predict the age-standardized incidence, mortality, and DALYs rate for NMSC by sex from 2022 to 2036[16]. The model allowed for the inference of unknown parameters by combining the posterior information obtained from the sample information with the a priori information on the unknown parameters[17], a process implemented using the BAPC and Integrated Nested Laplace Approximation (INLA) packages in R 4.3.2. To verify the accuracy of the model, we calculated the coefficient of determination (R²) for each BAPC model. R² reflects the extent to which the model explains the variation in the data. The closer the value is to 1, the better the model fit and the higher the prediction accuracy.”

Detailed comments:
**Abstract**
1. A comprehensive grammar and style revision is recommended. The section reads as a rough translation and would benefit from professional language editing or rewriting with better fluency.
“The increase rates (707.31%, 16.00%, and 10.04%) and upward trends -” The wording is unclear and needs to be clarified as to what these percentages refer to. A language and grammar check throughout the abstract is needed to improve readability and academic tone.

**Response:** Thank you very much for your valuable suggestions. We have revised and improved the expression of abstract.
2. The abstract mentions using the Joinpoint regression model and the Bayesian age-period-cohort model. Still, it does not specify how the data were handled, what population demographics were considered, or how the models were validated. A sentence on the scope of data (like population, age range) and model assumptions would improve transparency

**Response：**Thank you very much for your guidance. We have made modifications and supplements in lines 7-13: “Methods: Data related to the incidence, death, and disability-adjusted life years (DALYs) of NMSC in China and the world were obtained from the Global Burden of Disease (GBD) 2021 database. The population included in this sutdy were adults aged 20 and above. The Joinpoint regression model was used to estimate the average annual percentage of change (AAPC) to reflect the time trend. Bayesian age-period-cohort model was constructed for prediction, and the coefficient of determination R² was used to reflect the fitting degree of the model.”

3. In the results, are the percentages the total increase over the period or annual averages? How do the values compare between China and the global average? It is recommended to focus on a few key figures and interpret them succinctly in context.

**Response:** Thank you very much for your valuable suggestions. The percentage represents the overall trend change from 1990 to 2021. We have made some modifications to the results section in lines 14-24 and hope to receive your understanding: Results: From 1990 to 2021, the age-standardized incidence rate, mortality rate and DALYs rate of NMSC in China increased by 707.31%, 16.00% and 10.04%, respectively, all higher than the global level. And the corresponding upward trends were also more obvious, with AAPC of 6.71% (95%: 6.01% - 7.18%), 0.46% (95%: 0.40% - 0.52%) and 0.28% (95%: 0.22% - 0.34%), respectively. The incidence rate and its AAPC of NMSC were higher in men than in women. Moreover, the mortality rate and DALYs rate were also higher in men, but the growth rate was faster in women. The high incidence rate, mortality rate, and DALYs rate of NMSC all occurred in the higher age groups. The age-standardized incidence rates of NMSC in China and globally were predicted to continue to rise over the next 15 years, while the age-standardized mortality rates will decline.”

**Introduction**
1. Use the full term first with abbreviation (e.g., "disability-adjusted life years (DALYs)") and remain consistent in using “DALYs” instead of alternating between singular and plural forms.

**Response:** Thank you very much for your reasonable suggestions. We have revised the entire text.
2. Line 32: You mention "8% of all cancer deaths," but this conflicts with the relatively low mortality usually associated with NMSC. Double-check this figure.

**Response:** Thank you very much for your prompt discovery. We made the changes in lines 34-36: “According to GLOBOCAN, there were 1,234,595 new cases and 69,481 deaths of NMSC globally in 2022, accounting for 0.7% of all cancer deaths[2]”

3. Line 40: The statement “relatively few epidemiological studies on NMSC in China” is vague. You should briefly cite existing national-level epidemiological sources (if any) to support the claim, or clarify whether the lack is due to underreporting, registry limitations, or low research focus.

**Response:** Thank you very much for your reasonable suggestions. In the introduction section of the revised manuscript, lines 50-52, we have added more relevant supporting references: “Compared with melanoma, NMSC has a lower mortality rate and better prognosis. This difference has led to limited scientific evidence on the epidemiological characteristics and burden of NMSC in China[12].”

**Materials and Methods**
1. What ICD codes were used to define NMSC in the GBD dataset? (e.g., ICD-10 C44 for NMSC?) Were age-standardized rates obtained directly from GBD or calculated independently? Clarify the age-standardization process (e.g., using which standard population). What age groups were included in the study? How were male and female data handled? Were trends analyzed separately by sex for each metric? Cite the methods chosen by the previous literature and mention/quote your selection criteria for a specific test.

**Response:** Thank you very much for your reasonable suggestions. We have made revisions in lines 63-71: “Data related to the burden of NMSC were obtained from the GBD 2021 database, which provided epidemiological data for 204 countries or regions, 371 diseases, and 88 risk factors, and provided a strong basis for detailed and extensive understanding of global health trends and emerging challenges[13]. This study was based on the GBD 2021 database and used the C44 code in the International Classification of Diseases, Tenth Revision (ICD-10) to define NMSC[13]. This database provided the number of new cases, deaths, disability-adjusted life years (DALYs) and their corresponding rates of NMSC for population aged 20 and above, categorized by different genders[13].”

In lines 73-82: “Using the GBD 2021 world standard population structure, this study applied the direct method to age-standardise the incidence, mortality, and DALYs rates of NMSC in China and globally. This method was based on the assumption that these indicators followed an independent Poisson random variable weighted sum distribution. By controlling for changes in population age structure, it accurately quantified differences in the burden of NMSC across different time periods, genders, and regions. The calculation formula was as follows:

$$\text{Age−standardized rate=}\frac{\sum_{\text{i=1}}^{\text{A}} \text{a}_{\text{i}}\text{W}_{\text{i}}}{\sum_{\text{i=1}}^{\text{A}} \text{W}_{\text{i}}}$$

Where ai indicates the age-specific rate for the i-th age group, Wi indicates the weight of the corresponding age subgroup in the selected reference population (i represents the i-th age category), and A indicates the total number of age groups.”

2. The model should be described more formally: Did you use the National Cancer Institute’s Joinpoint Regression Program? Specify the full name and source for software: “Joinpoint Regression Program (version 5.0.2) developed by the U.S. National Cancer Institute.”

**Response:** Thank you very much for your reasonable suggestions. We have made revisions and additions to lines 115-119: “This study used the Joinpoint Regression Program (version 5.0.2) developed by the U.S. National Cancer Institute to perform Joinpoint regression analysis. Graphical visualization was conducted using R software (version 4.3.2, https://www.r-project.org/), and the BAPC and INLA packages were utilized to construct the BAPC model.”

3. What significance level was used to identify joinpoints? Were there any constraints on the number of joinpoints? Was model selection based on a permutation test?

**Response:** Thank you very much for your guidance and suggestions. We have made the following additions in lines 84-99: “The Joinpoint regression model was a log-linear model with time (year) as the independent variable and incidence or mortality as the dependent variable[14]. It described the trend of incidence or mortality by splicing the joinpoints of different logarithmic line segments[15]. This model established segmented regression based on the temporal characteristics of disease distribution, dividing the study period into different intervals using multiple connection points, and fitting and optimizing the trends within each interval to assess the characteristics of specific disease changes within different intervals[14]. During model construction, Monte Carlo simulation tests were used to determine the number of connection points, their positions, and corresponding P-values. A two-sided test was employed with a significance level of 0.05, and the Bonferroni method was applied to correct the significance level and control the false positive rate[14]. In this study, the number of joint points was set within the range of 0 to 6 to ensure the model adequately captures temporal trend changes while avoiding overfitting. The model was used to calculate the average annual percentage change (AAPC) and its 95% confidence interval (CI) to reflect the trend over time. The model formula was as follows[14]”

4. In the Bayesian age-period cohort model, what R package was used? What were the prior assumptions, time intervals, or model parameters? How was model fit assessed?This section is incomplete without including BAPC modeling details, which are critical for prediction validity.

**Response：**Thank you very much for your reasonable suggestions. We have made corresponding revisions in lines 105-113: “We used the BAPC model to predict the age-standardized incidence, mortality, and DALYs rate for NMSC by sex from 2022 to 2036[16]. The model allowed for the inference of unknown parameters by combining the posterior information obtained from the sample information with the a priori information on the unknown parameters[17], a process implemented using the BAPC and Integrated Nested Laplace Approximation (INLA) packages in R 4.3.2. To verify the accuracy of the model, we calculated the coefficient of determination (R²) for each BAPC model. R² reflects the extent to which the model explains the variation in the data. The closer the value is to 1, the better the model fit and the higher the prediction accuracy.”
**Results**
1. Lines 99-103: The last part about age groups is confusing and partially contradictory. You say: “the lowest was 85+ years old,” then shortly after, say “highest in the world was 85+ years.” Also, 20–54 years is described as the lowest age-specific incidence, but that contradicts earlier groupings. Clarify this.

**Response:** Thank you very much for your careful review. We failed to clearly distinguish between the number of cases and the incidence rate. We are very sorry and have made the correction in lines 137-144: “Both in China and the world, the number of cases, incidence rate, ASIR, as well as the corresponding increase rate and AAPC of NMSC were all higher in men than in women. In China, the age group with the highest number of NMSC cases was 65-74 years old, and the lowest was over 85 years old. Globally, the age group with the highest number of cases was 65-74 years old, and the lowest was 55-59 years old. The age group with the lowest incidence rate in both China and globally was 20-54 years old. The age group with the highest incidence rate in China was 80-84 years old, while globally it was those above 85 years old. (Table 1, Figure 1)”
2. Lines 141-148: While the data is informative, there’s little attempt to interpret or explain what these DALY trends might mean (e.g., differences in disability impact, treatment access, demographic effects).

**Response:** We fully agree with this view. DALYs is a comprehensive indicator that can fully reflect the disease burden, including the loss of life due to premature death (YLLs) and the loss of healthy life due to disability (YLDs). We conducted additional discussions in the discussion section, analyzing multiple factors that might be reflected in the trend changes of NMSC's DALYs, including differences in the allocation of medical resources and accessibility of treatment among different regions, the impact of population aging on the disease burden, and the potential influence of environmental factors and lifestyle changes on the epidemiological characteristics of diseases. These discussions help to gain a deeper understanding of the social and public health significance behind the changes in the disease burden of NMSC. We made corresponding revisions in lines 272-281: “Based on this trend, it is recommended to strengthen the early screening and diagnosis system for NMSC, especially in areas with scarce medical resources; conduct health education and regular check-ups for key populations such as the elderly and young women; incorporate NMSC prevention and control into climate change response strategies and strengthen the construction of UV radiation warning systems; optimize the allocation of medical resources to enhance primary healthcare facilities' diagnostic and treatment capabilities; more national and subnational epidemiological studies on NMSC should be conducted to establish prevention and control strategies and clinical guidelines tailored to the national context, providing scientific evidence for public health decision-making.”
**Discussion**
1. The discussion blends a summary of results, external context, and policy implications without clear thematic organization. It begins with a generic overview and then jumps to specific issues like tanning, ozone depletion, and tourism without adequate linkage. There’s a lack of focused structure. The key finding that China has a lower incidence but faster increases and relatively high DALY/mortality burdens deserves deeper analysis. Is this due to late diagnosis, limited access to dermatologic care, or underreporting of non-lethal cases? Why are DALYs and deaths rising faster than globally, despite lower baseline rates?Statements like “this may be related to…” or “it has been shown that…” are too vague. Citations [14] to [17] are not explained or integrated. It is unclear what study [14] found and how it aligns or differs from your own results. Clearly describe each cited study and explicitly link it to your findings.

**Response：**Thank you very much for your crucial suggestions. We have improved the discussion section and hope to receive your approval. We have supplemented the findings of the 14th reference (which is now the 18th) to facilitate the connection with our results. At the same time, we have also explained why the mortality rate and DALYs in China are higher. If there are still any imperfections, please continue to ask questions and we will actively make some revisions.

We have made corresponding revisions in lines 235-271: “The burden of NMSC increased in China and globally from 1990 to 2021, and the increase rate in China was higher than the global level. This finding was consistent with another study, which indicated that between 1992 and 2021, China showed an upward trend in NMSC mortality rate, which remained higher than the global average[18]. This suggested that the NMSC continued to be a significant public health concern that cannot be overlooked. This may be influenced by multiple factors, including population aging, environmental pollution, ozone layer depletion, and climate change. As a high-risk group for NMSC, the global trend of an increasing proportion of elderly people had significantly impacted the incidence and mortality rates of this disease[19]. Additionally, UV radiation, a known risk factor for NMSC, played a key role in the pathogenesis of skin cancer by causing DNA damage and immunosuppression[5]. Climate change had caused various health issues worldwide, from heat-related illnesses to changes in the geographical distribution of infectious diseases and increased risks of zoonotic disease transmission, with its impact on human health becoming increasingly widespread[20]. Among these climate change-related health threats, the incidence of NMSC increased significantly, becoming one of the key manifestations of climate change's impact on human health[21]. Climate change accelerated ozone layer depletion, increasing surface UV radiation intensity and raising human exposure to UV radiation[22]. Additionally, socio-economic development and lifestyle changes exacerbated this issue. As economic levels improved, people had more opportunities to participate in outdoor activities and vacation travel, increasing the likelihood of sunburn. Sunburn was significantly associated with an increased risk of NMSC, particularly among women under 25. These factors intertwine, collectively contributing to the ongoing global rise in the burden of NMSC[23]. It is worth noting that during this period, compared to the global level, the incidence of NMSC in China was relatively low but increasing rapidly, while the mortality and DALYs were relatively high. This may be related to multiple factors. For instance, late diagnosis and uneven distribution of medical resources could be important reasons. Studies have shown that NMSC is often overlooked, leading to delayed diagnosis[11]. Underreporting of non-fatal cases is also a possible factor. Compared to other cancers with high mortality rates (such as lung cancer, gastric cancer, or pancreatic cancer), NMSC had a relatively low mortality rate. This may lead to healthcare resources and public health attention being directed more toward those more lethal cancer types. Due to the relatively low baseline incidence of NMSC in China, the healthcare system did not place sufficient emphasis on this disease, resulting in incomplete reporting of non-fatal NMSC cases[24].”

2. Consider gender-specific behaviors (e.g., tanning practices, occupational exposures, cosmetic trends), and whether care-seeking or diagnosis patterns differ. Expand discussion beyond UV exposure. Consider biological factors (e.g., hormone-related differences in immune response), or differences in early detection and healthcare access. Consider referencing recent studies on sex-specific tumor behavior, if available, or national screening trends. Use established concepts like cumulative UV exposure, immunosenescence, or actinic damage to describe the mechanisms more accurately. Please see Sarwar Z., Nomi Z.A., Awais M., Shahbakht R.M., et al. (2025) for a broader discussion on the impact of climate change on disease patterns, including environmental triggers such as increased UV exposure that may contribute to the rising burden of skin-related diseases. Effect of Climate Change on Transmission of Livestock Diseases. Agrobiological Records, 19, 1–11. https://doi.org/10.47278/journal.abr/2025.001

**Response：**Thank you very much for your reasonable and valuable suggestions. We have added information about the impact of climate based on your advice, and have cited the literature you provided. We are very touched by this and would like to thank you for your assistance. We have made corresponding revisions in lines 240-281: “This may be influenced by multiple factors, including population aging, environmental pollution, ozone layer depletion, and climate change. As a high-risk group for NMSC, the global trend of an increasing proportion of elderly people had significantly impacted the incidence and mortality rates of this disease[19]. Additionally, UV radiation, a known risk factor for NMSC, played a key role in the pathogenesis of skin cancer by causing DNA damage and immunosuppression[5]. Climate change had caused various health issues worldwide, from heat-related illnesses to changes in the geographical distribution of infectious diseases and increased risks of zoonotic disease transmission, with its impact on human health becoming increasingly widespread[20]. Among these climate change-related health threats, the incidence of NMSC increased significantly, becoming one of the key manifestations of climate change's impact on human health[21]. Climate change accelerated ozone layer depletion, increasing surface UV radiation intensity and raising human exposure to UV radiation[22]. Additionally, socio-economic development and lifestyle changes exacerbated this issue. As economic levels improved, people had more opportunities to participate in outdoor activities and vacation travel, increasing the likelihood of sunburn. Sunburn was significantly associated with an increased risk of NMSC, particularly among women under 25. These factors intertwine, collectively contributing to the ongoing global rise in the burden of NMSC[23]. It is worth noting that during this period, compared to the global level, the incidence of NMSC in China was relatively low but increasing rapidly, while the mortality and DALYs were relatively high. This may be related to multiple factors. For instance, late diagnosis and uneven distribution of medical resources could be important reasons. Studies have shown that NMSC is often overlooked, leading to delayed diagnosis[11]. Underreporting of non-fatal cases is also a possible factor. Compared to other cancers with high mortality rates (such as lung cancer, gastric cancer, or pancreatic cancer), NMSC had a relatively low mortality rate. This may lead to healthcare resources and public health attention being directed more toward those more lethal cancer types. Due to the relatively low baseline incidence of NMSC in China, the healthcare system did not place sufficient emphasis on this disease, resulting in incomplete reporting of non-fatal NMSC cases[24].

Based on this trend, it is recommended to strengthen the early screening and diagnosis system for NMSC, especially in areas with scarce medical resources; conduct health education and regular check-ups for key populations such as the elderly and young women; incorporate NMSC prevention and control into climate change response strategies and strengthen the construction of UV radiation warning systems; optimize the allocation of medical resources to enhance primary healthcare facilities' diagnostic and treatment capabilities; more national and subnational epidemiological studies on NMSC should be conducted to establish prevention and control strategies and clinical guidelines tailored to the national context, providing scientific evidence for public health decision-making.”

3. Include mention of improved early detection, increased awareness, and treatment advances (as you briefly touch on with the melanoma example). Clarify whether such progress applies to NMSC specifically. The example about melanoma treatments (e.g., checkpoint inhibitors) may not apply unless explicitly linked. For integrating plant-based compounds into zoonotic disease control strategies, particularly for their potential anthelmintic and immunomodulatory effects, please see Alsayeqh, A.F. (2025). Botanicals: A Promising Control Strategy Against Highly Zoonotic Foodborne Trichinosis. Kafkas Üniversitesi Veteriner Fakültesi Dergisi, 31(2), 129–136. https://doi.org/10.9775/kvfd.2023.32154

**Response:** Thank you very much for your suggestions and help. We have made corresponding revisions in lines 328-339: “Between 2013 and 2017, the introduction of immune checkpoint inhibitors and targeted therapies significantly reduced melanoma mortality rates in the United States[38–40]. Similar therapeutic advancements were gradually applied to NMSC. For example, the PD-1 inhibitor Cemiplimab had been approved by the FDA for advanced cutaneous squamous cell carcinoma, with an objective response rate of up to 47%[41]; the Hedgehog pathway inhibitor Vismodegib achieved an objective response rate of 43% in advanced basal cell carcinoma[42]. Access to these therapies in China is gradually improving, such as the clinical trials of the domestically produced PD-1 inhibitor Toripalimab in skin cancer, and the widespread adoption of early diagnostic technologies (such as dermatoscopy and AI-assisted screening)[43–46], which may drive a reduction in mortality rates and disease burden for NMSC in China.”

4. Include one or two concrete examples of relevant NMSC-related actions under the policy, or state the lack thereof. The section contains meaningful points but needs a more precise, evidence-backed explanation of observed trends, especially regarding aging, gender, and future burden. It should also clarify distinctions between NMSC and melanoma, and better integrate policy and prevention contexts.

**Response:** Thank you very much for your suggestion. We have made corresponding revisions in lines 345-373: “To address the rising trend in the burden of NMSC, China and other countries implemented a series of targeted policy interventions. Environmental pollution has been proven to impact skin cancer significantly[47]. To this end, China's Environmental Protection Law, which came into effect on January 1, 2015, provided legal protection for reducing the burden of NMSC by strictly limiting the concentration of environmental pollutants that may increase the risk of NMSC[18]. Through measures such as clean production and pollutant emission control, this law will reduce environmental factors that may increase the risk of NMSC from the source, complementing improvements in healthcare and disease prevention strategies to collectively establish a multi-dimensional policy framework for NMSC prevention and control in China. At the global level, a series of skin cancer prevention and control projects and campaigns focused on reducing the incidence of skin cancer through primary prevention measures such as health education and reducing exposure[48]. Notable initiatives included the “SunPass” project launched by the European Skin Cancer Foundation in 2009 [49] and the “SunSmart” campaign initiated by the UK Cancer Research Center in 2003[50]. These programs effectively enhanced public awareness of skin cancer prevention through systematic public education, sun protection knowledge dissemination, and intervention in risky behaviors, thereby reducing the risk of skin cancer at its source. As global climate change and environmental pollution persist, NMSC prevention and control will face new challenges. In the future, countries must strengthen policy coordination, integrate environmental protection and public health strategies, and improve monitoring and early warning systems. Additionally, artificial intelligence and large data technologies should be utilized to enhance the accuracy of early cancer screening, innovative technologies and methods should be applied to cancer treatment, and to develop personalized prevention plans and treatment strategies[51–53]. Strengthening international cooperation and sharing prevention and control experiences are crucial for building a global NMSC prevention and control network and reducing the disease burden.”
